# Genome-Wide Identification and Water Stress Response of the *WRKY* Gene Family in *Annamocarya sinensis*: An Endangered Plant with an Extremely Small Population

**DOI:** 10.3390/biology15161429

**Published:** 2026-08-19

**Authors:** Youxue Chen, Shengjie Sun, Dan Li

**Affiliations:** 1 College of Forestry, Southwest Forestry University, Kunming 650224, China; chenyouxue2026@163.com; 2 Laboratory of Yunnan Institute of Biodiversity, Southwest Forestry University, Kunming 650224, China; 3 State Key Laboratory of Tree Genetics and Breeding, Binhai Forestry Research Center, Research Institute of Forestry, Chinese Academy of Forestry, Beijing 100091, China

**Keywords:** *Annamocarya sinensis*, *WRKY* gene family, bioinformatics analysis, water stress, expression analysis

## Abstract

*Annamocarya sinensis* is a Plant Species with Extremely Small Populations (PSESP) of great ecological and research value. Water conditions serve as a key environmental factor that profoundly shapes its growth, physiological homeostasis and stress adaptation. WRKY transcription factor families constitute the core molecular regulatory modules orchestrating plant responses to water-related stresses and environmental fluctuations. This study performs a systematic identification and bioinformatics analysis of the *WRKY* gene family in *A. sinensis*, combined with RT-qPCR to verify and analyze its expression patterns under water stress. The results identified a total of 92 *WRKY* genes unevenly distributed across 16 chromosomes, classified into three distinct subfamilies with obvious conservation of conserved motifs; the gene promoters were enriched with drought cis-elements, and fragment duplication drove the expansion of the family; this family had a high collinearity with the homologous genes of *Arabidopsis thaliana*, and its evolutionary function was conserved. RT-qPCR confirmed that *AsWRKY3*, *AsWRKY11*, *AsWRKY17*, *AsWRKY53* and *AsWRKY72* showed differential expression under water stress and were involved in regulating the processes of drought and waterlogging tolerance. Under drought stress, all five genes exhibited an upregulation trend, albeit with slightly varying response intensities; under waterlogging stress, *AsWRKY3* and *AsWRKY17* demonstrated strong enhanced responses, *AsWRKY11* and *AsWRKY53* showed early-response patterns, whereas *AsWRKY72* exhibited a downregulation trend. In summary, fragment duplication and family differentiation enhanced the functional diversity of the *WRKY* family, providing a genetic basis for the adaptation of *A. sinensis* to changes in water availability. The research findings not only provide crucial theoretical clues for exploring the potential environmental adaptation and molecular evolution mechanisms of extremely small population plants, but also offer essential genetic resources and scientific foundations for species conservation, restoring wild populations, and conducting stress-resistant molecular breeding.

## Simple Summary

*Annamocarya sinensis* exhibits growth and physiological traits that are highly responsive to variations in water supply. Plants synthesize a wide range of specialized proteins to coordinate growth and cope with unfavorable water conditions, and WRKY transcription factors represent a core class of these regulatory proteins. Although WRKY transcription factors have been well documented in many plant species, the regulatory patterns of *WRKY* genes under water stress have not been systematically characterized in *A. sinensis*. This study systematically identifies and analyzes the *WRKY* gene family in this species at the whole-genome level, and investigates their expression patterns under water stress. We found that *WRKY* genes are unevenly distributed across chromosomes and can be classified into three major groups; they contained functional elements that help plants adapt to unfavorable conditions, such as drought-responsive elements. The *WRKY* genes identified in *A. sinensis* showed genetic homology with those in *Arabidopsis thaliana*. Furthermore, we discovered five *WRKY* genes that may enhance or suppress plant responses to changes in water availability. This study provides insights into the evolutionary history and potential functional mechanisms of *WRKY* genes in *A. sinensis* and Plant Species with Extremely Small Populations, offering valuable data for breeding drought- and waterlogging-tolerant, stress-resistant varieties.

## 1. Introduction

**Figure 1:**
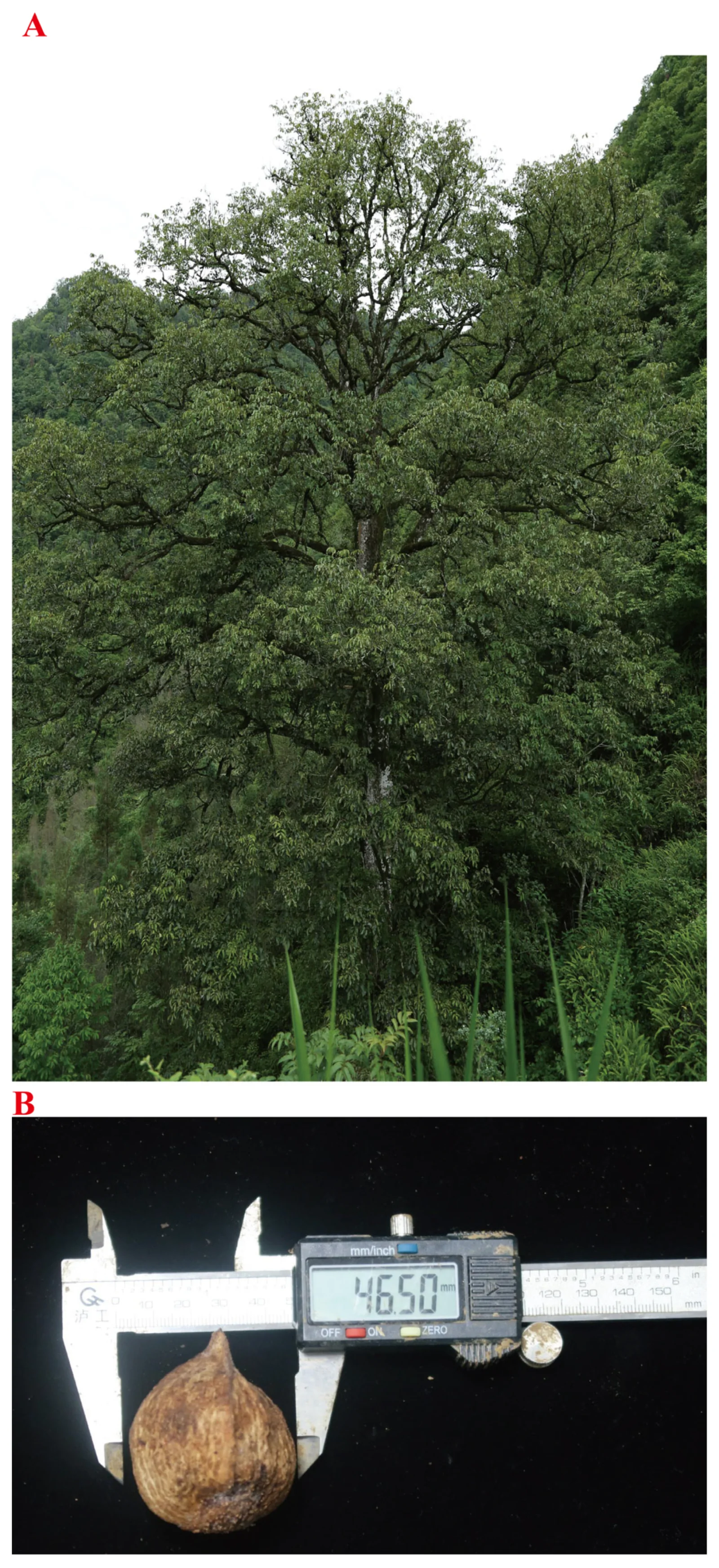
Adult tree and fruit of *A. sinensis.* (**A**) above shows an adult *A. sinensis* tree; (**B**) below shows the fruit.

*Annamocarya sinensis* is a plant belonging to the Juglandaceae family and the *Annamocarya* genus; it is considered a relic plant from the Tertiary period that survived in the ancient tropical regions, occupies a distinctive phylogenetic position within Juglandaceae and shows divergent evolutionary features compared with its close relatives [1]. It can be readily distinguished from other Juglandaceae species by the distinct acuminate beak at the apex of its nut endocarp, and mature individuals develop large stature (Figure 1). *A. sinensis* has extremely high utilization value: its bark and outer fruit peel can be used to extract tannins, the fruit shells can be made into activated carbon, the seeds have a relatively high oil content, and the wood is commonly used for furniture and musical instruments, with wide applications in food and industry [2]. *A. sinensis* has a narrow distribution range and is currently found only in southern China, including a few regions such as Yunnan and Guizhou provinces, as well as northern Vietnam [3]. Poor natural regeneration, narrow endemic distribution, and extreme susceptibility to hydrological fluctuations collectively limit its wild population to an extremely small size, qualifying the species as a Class II national protected wild plant in China [4]. In the “*IUCN Red List of Threatened Species*”, its assessment level is Endangered (EN). Exploring the potential mechanisms underlying the response of *Annamocarya sinensis* to water stress can help interpret its endangered status and provide a theoretical basis for targeted habitat restoration, population recovery, and conservation of wild germplasm resources.

As core functional factors regulating gene expression, transcription factors play a pivotal role in plant growth, development and stress response mechanisms [5,6]. The *WRKY* gene family is one of the largest transcription factor families in higher plants, involved in the synthesis of carbohydrates and secondary metabolites [7]. It also plays a crucial role in the growth, development and aging of plants [8,9]. The proteins of the WRKY transcription factor family contain a highly conserved 60-amino-acid WRKY domain, which features a conserved WRKYGQK core sequence at its N-terminus and a zinc finger motif (composed of cysteine and histidine residues within the WRKY domain) at its C-terminus, enabling specific recognition of the W-box cis-element (TTGACC/T) in target gene promoters [9,10]. Based on the differences in the number of WRKY domains and zinc finger domains, this family is divided into three major categories [11]. Functional studies conducted in model plants such as *Arabidopsis thaliana* and *Oryza sativa* have revealed that WRKY transcription factors are key regulators of non-biological stress responses (such as drought, high temperature, low temperature, and salinity) [12]. Functional analyses of *WRKY* genes have been performed in a wide range of woody plants such as *Populus* and *Pinus*. Meanwhile, similar research has also been conducted in multiple economic tree species, including *Zanthoxylum bungeanum* Maxim and *Juglans regia.* However, there are currently no systematic studies on the *WRKY* gene family in *A. sinensis*.

Water deficit and waterlogging represent major abiotic constraints limiting the growth of subtropical woody plants. Drought and waterlogging induce divergent physiological and transcriptional responses in trees; however, most investigations focus on plant adaptation to either drought or waterlogging alone, and comparative analyses exploring both stresses simultaneously remain limited [13,14]. WRKY transcription factors are key regulators of plant abiotic stress tolerance. Although WRKY responses to water stress have been documented in many broad-leaved tree species, relevant research on the endangered relict species *Annamocarya sinensis* is still lacking. In this study we combined bioinformatics analysis with gene expression patterns to systematically identify the members of the *WRKY* gene family in the *A. sinensis* genome. We conducted a comprehensive analysis of their physicochemical properties, gene structure, evolutionary relationships, conserved motifs, cis-regulatory elements, colinearity, and gene expression patterns in response to water stress. This study systematically elucidated the characteristics of the *WRKY* gene family in *A. sinensis* and its response patterns under water stress, and provided reliable experimental data and theoretical foundations for exploring the water stress response mechanisms of *A. sinensis* and conserving its wild populations.

## 2. Materials and Methods

### 2.1. Materials

Nine half-sib families of *A. sinensis* with similar biological characteristics were selected as experimental materials.

### 2.2. Methods

#### 2.2.1. Water Stress Treatment of *A. sinensis*

Plants were randomly divided into three groups, with three biological replicates for each treatment group. The control group (CK1–CK3) received adequate water supply with daily watering. The drought stress group (D1–D3) experienced water deprivation, with a rubber sheet placed beneath the pot to prevent contact with the moist soil. The waterlogging stress groups (W1–W3) were placed in buckets with water levels just above the soil surface, and the water was replaced daily. Based on the plant’s response characteristics to water stress, drought treatment was imposed by withholding irrigation for 20 days [13]. Leaf relative water content (RWC) was determined to verify the establishment of drought stress. Waterlogging treatment lasted for 40 days, according to experimental protocols for Juglandaceae species [14]. Visible leaf wilting was observed during treatment, demonstrating successful imposition of waterlogging stress [15,16]. Every five days, leaves from the control group, drought-stressed group, and waterlogging-stressed group of *A. sinensis* plants were harvested. The leaves were washed on the plants with 75% alcohol by volume, then rapidly frozen in liquid nitrogen after the alcohol had evaporated, and stored at −80 °C for later use.

#### 2.2.2. Identification and Physicochemical Property Analysis of *A. sinensis* WRKY Gene Family

Using known protein sequences from *Arabidopsis thaliana* as references, we retrieved their protein sequences, whole-genome files, and annotation files from the TAIR database (https://www.arabidopsis.org/, accessed on 7 June 2025). The genome sequencing of *A. sinensis* was obtained from the Juglandaceae Genome Projects (https://cmb.bnu.edu.cn/juglans/, accessed on 7 June 2025); the hidden Markov model file for the WRKY transcription factor (PF03106) was obtained from the Pfam database (https://www.uniprot.org/database/DB-0073, accessed on 7 June 2025); and the reference genome file of *A. sinensis* was downloaded from the Juglandaceae Genome Projects database (https://cmb.bnu.edu.cn/juglans/, accessed on 7 June 2025) [17]. Candidate WRKY proteins were screened using two complementary approaches implemented with TBtools-II v2.472. Homology searches were performed via BLAST (wrapped within TBtools-II v2.472), with an E-value cutoff of 1 × 10^−5^. Conserved domain screening was conducted simultaneously using the profile HMM. Results obtained from the two approaches were combined, and redundant sequences were removed. All candidate proteins were manually examined, and sequences lacking intact WRKY conserved domains were excluded to obtain the final set of *AsWRKY* family members [18]. The identified *WRKY* genes were named sequentially according to their positions on chromosomes. The physicochemical parameters of *AsWRKY* family members, including amino acid number (aa), molecular weight (MW), theoretical isoelectric point (pI), instability index, aliphatic index (AI), and grand average of hydropathicity (GAP), were calculated and analyzed using the Protein Parameter Calc (ProtParam-based) module in TBtools software [19,20].

#### 2.2.3. Chromosomal Localization and Collinearity Analysis of *A. sinensis* WRKY Gene

The chromosomal localization of the *AsWRKY* gene family was visualized using the Gene Location Visualize from GTF/GFF function in TBtools software, based on *A. sinensis* genome annotation file (GFF). Intraspecific collinearity analysis of *A. sinensis* was performed using the One Step MCScanX-SuperFast module, and interspecific collinearity files were obtained via BLAST alignment *Arabidopsis*. Intra-species and inter-species collinearity *WRKY* genes were subsequently generated [21].

#### 2.2.4. Systematic Evolution, Conserved Motifs, and Promoter Analysis in *A. sinensis* WRKY Gene Family

Sequences were imported into MEGA 11 software for multiple sequence alignment of protein sequences from *A. sinensis* and *Arabidopsis* target genes. A phylogenetic tree was constructed using the neighbor-joining (NJ) method with the Poisson substitution model. Gaps and missing data were treated by pairwise deletion, and 1000 bootstrap replicates were used to evaluate the reliability of tree topology [21,22]. The selected protein sequences were submitted to the NCBI Batch CDD database for CD-search, and the candidate sequences were uploaded to the MEME website. MEME was then used to identify conserved motifs in members of the WRKY transcription factor family, with a maximum of six motifs allowed, and the motif width was limited to 6–50 amino acids. All members were checked for the presence of complete WRKY structural domains. The 2000 bp sequences upstream of the translation initiation codon (ATG) of each *WRKY* gene in *A. sinensis* were retrieved as promoter sequences using TBtools. Cis-acting regulatory elements within these promoter regions were predicted using PlantCARE (https://bioinformatics.psb.ugent.be/webtools/plantcare/html/, accessed on 8 June 2025) online database [18].

#### 2.2.5. Expression Analysis of *A. sinensis* WRKY Gene Family Under Water Stress

To analyze the expression response characteristics of *WRKY* gene family members in *A. sinensis* under water stress, the relative water content of leaves was measured under drought stress conditions. *Actin* was selected as the reference gene for RT-qPCR expression analysis. Studies on *Arabidopsis thaliana* and woody plant species have verified that *Actin* possesses stable transcriptional levels in vegetative tissues exposed to diverse abiotic stresses, supporting its adoption as an internal control in this study [23,24,25]. Multiple criteria were integrated to screen candidate genes, including transcriptomic expression patterns under drought and waterlogging treatments, conserved structural features of encoded proteins, phylogenetic clustering, functional annotations of homologous genes in woody plant species, and syntenic relationships with *Arabidopsis thaliana*. Genes showing remarkable transcriptional responses to water stress were prioritized for quantitative validation (detailed sequence information can be found in Table 1). The RT-qPCR method was employed for detection and verification. The control, drought, and waterlogging groups included three biological replicates. For RT-qPCR analysis, each cDNA template was amplified in three technical replicate wells. The control group, drought group, and waterlogging groups included three biological replicates. For RT-qPCR analysis, each cDNA template was amplified in three technical replicate wells. Ordinary one-way analysis of variance (ANOVA) was used to evaluate intergroup differences, and Bonferroni’s test was applied for post hoc pairwise comparisons. All statistical analyses were performed using GraphPad Prism 10. The relative expression levels of the genes were calculated using the 2^−ΔΔCt^ method [26]. Finally, data statistics and graphing were carried out.

## 3. Results

### 3.1. Identification and Physicochemical Property Analysis of A. sinensis WRKY Gene Family

Using a hidden Markov model based on the WRKY domain (PF03106), the genome sequences of *A. sinensis* were aligned with those of *Arabidopsis*. After two rounds of screening and removal of duplicates, a total of 92 members of gene families were identified. Physicochemical analysis of proteins encoded by the *WRKY* gene family revealed that their lengths ranged from 151 aa (*AsWRKY88*) to 771 aa (*AsWRKY78*), with molecular weights varying between 17.23 kDa and 86.92 kDa, indicating significant structural diversity among members of this family (detailed in Table 2). Their theoretical isoelectric points ranged from 5.01 (*AsWRKY91*) to 9.97 (*AsWRKY83*), with 50 proteins classified as acidic proteins (pI < 7) and 42 alkaline proteins (pI > 7); the proportion of acidic proteins (50/92) was greater than that of alkaline proteins. The instability indices ranged from 26.87 (*AsWRKY83*) to 67.26 (*AsWRKY38*). With the exception of *AsWRKY17* and *AsWRKY83*, whose instability indexes were below 40, all other proteins exhibited instability indices exceeding 40, indicating that the vast majority are unstable proteins. The aliphatic index ranged from 42.86 (*AsWRKY86*) to 72.48 (*AsWRKY78*). The average hydrophilicity value ranged from −1.199 (*AsWRKY88*) to −0.445 (*AsWRKY50*). All 92 family members were hydrophilic proteins, a trait closely linked to the capacity of this gene family to respond to water stress (detailed in Table S1).

### 3.2. Chromosomal Localization of A. sinensis WRKY Gene and Intraspecific Collinearity Analysis

**Figure 2:**
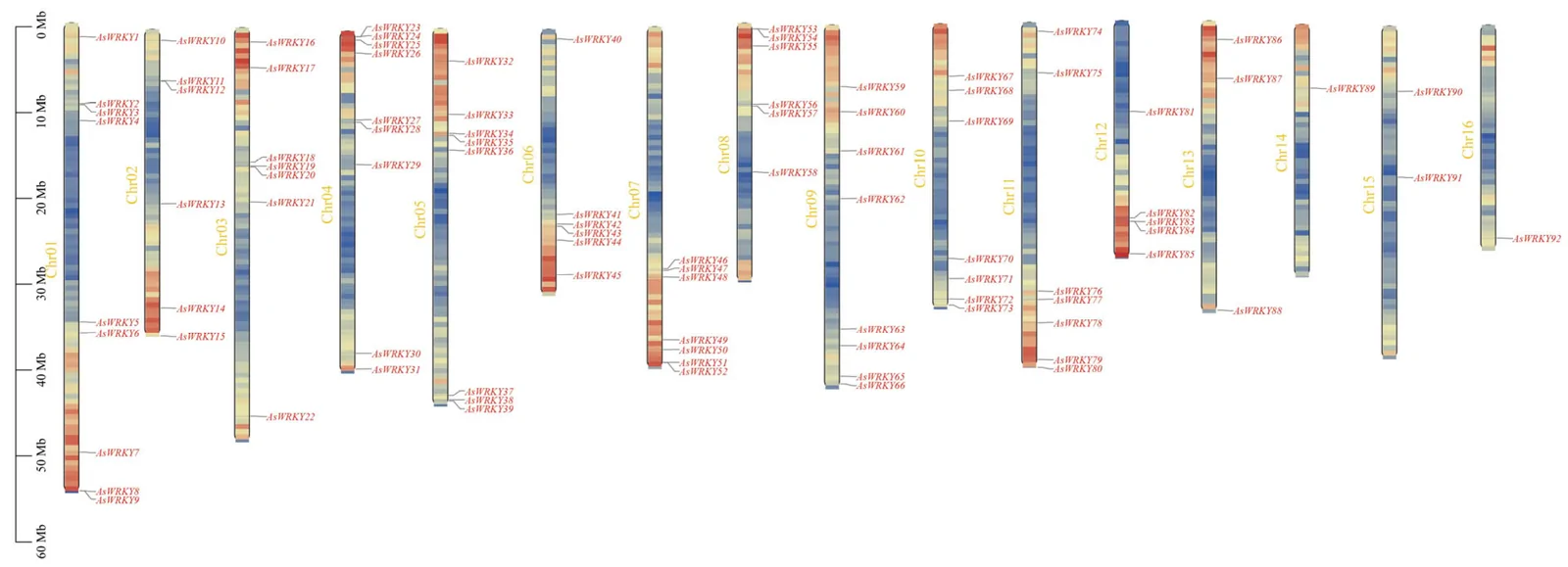
Chromosomal distribution of *AsWRKY* gene family members.

Based on their chromosomal locations, the 92 family members were designated as *AsWRKY1* to *AsWRKY92* (Figure 2). The results demonstrate that the *AsWRKY* genes are present on all 16 chromosomes, indicating widespread distribution of this gene family across the genome. Furthermore, the 92 *AsWRKY* genes are unevenly distributed across the 16 chromosomes of *A. sinensis.* Among them, chromosomes Chr01 and Chr04 contained the highest number of *AsWRKY* genes (nine in total), followed by Chr05 and Chr09 with eight genes each; chromosomes Chr14 and Chr16 had the lowest number at only one gene. Chromosomes Chr03, Chr07, Chr10, and Chr11 each contained seven genes, while chromosomes Chr02, Chr06, and Chr08 each had six genes. Chromosomes Chr12, Chr13 and Chr15 contained five, three, and two genes respectively. The majority of genes tended to be distribute in regions with higher density, with clustered gene distributions observed on chromosomes such as Chr04 and Chr05, indicating that tandem repeat events may be the primary driver of *AsWRKY* gene expansion and play a crucial role in responding to both biological and abiotic stresses.

**Figure 3:**
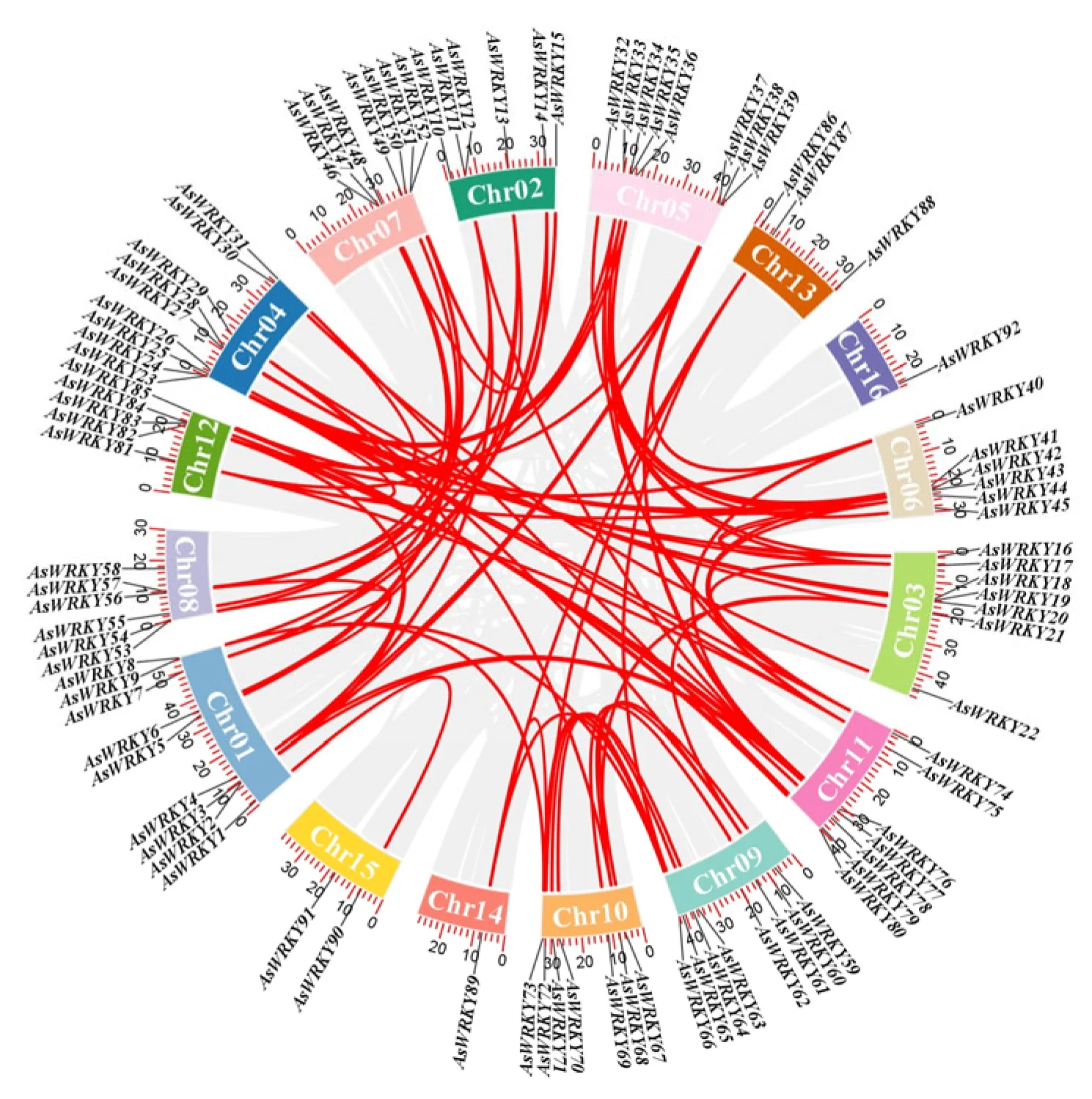
Collinearity analysis of *WRKY* genes in *A. sinensis*.

To further investigate the expansion and evolution of the *WRKY* gene family in *A. sinensis*, in this study we performed phylogenetic analysis on 92 *WRKY* genes within the species (Figure 3). The results demonstrated that, with the exception of *AsWRKY92* on chromosome 16, which lacked a collinearity relationship, all 15 other chromosomes exhibited such relationships, albeit unevenly distributed. Numerous intrafamilial segmental duplication events were identified among family members; for example, a significant segmental duplication occurred between *AsWRKY1* on chromosome Chr01 and *AsWRKY90* on chromosome Chr15, forming a pair of homologous conserved genes. The abundant interchromosomal linkages shown in the figure revealed that segmental duplication acted as the major driving force for the expansion of the *WRKY* gene family in *A. sinensis* and constituted the core mechanism responsible for genetic and functional divergence.

### 3.3. Systematic Evolution and Promoter Analysis in A. sinensis WRKY Gene Family

**Figure 4:**
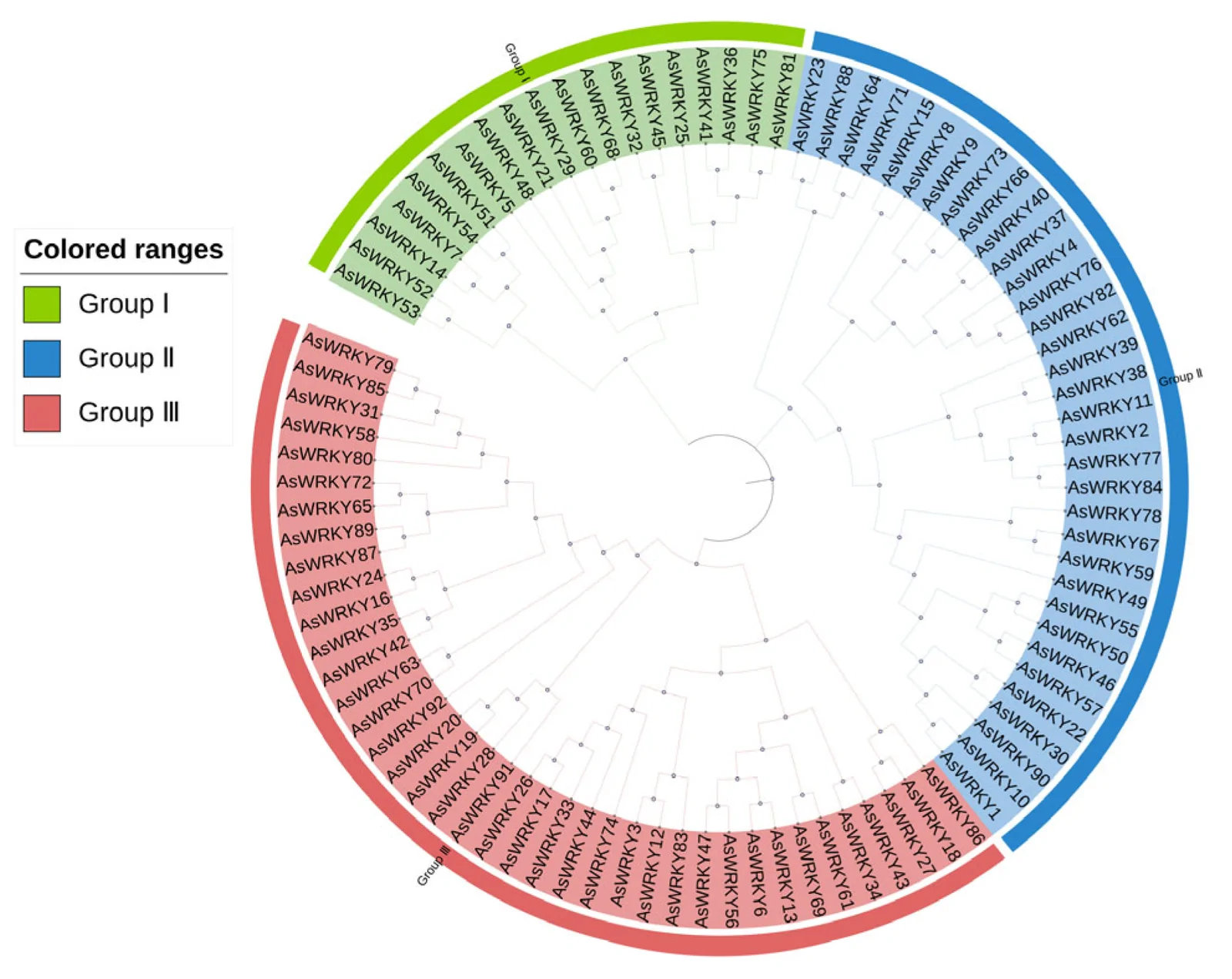
Phylogenetic tree of *WRKY* gene family.

**Figure 5:**
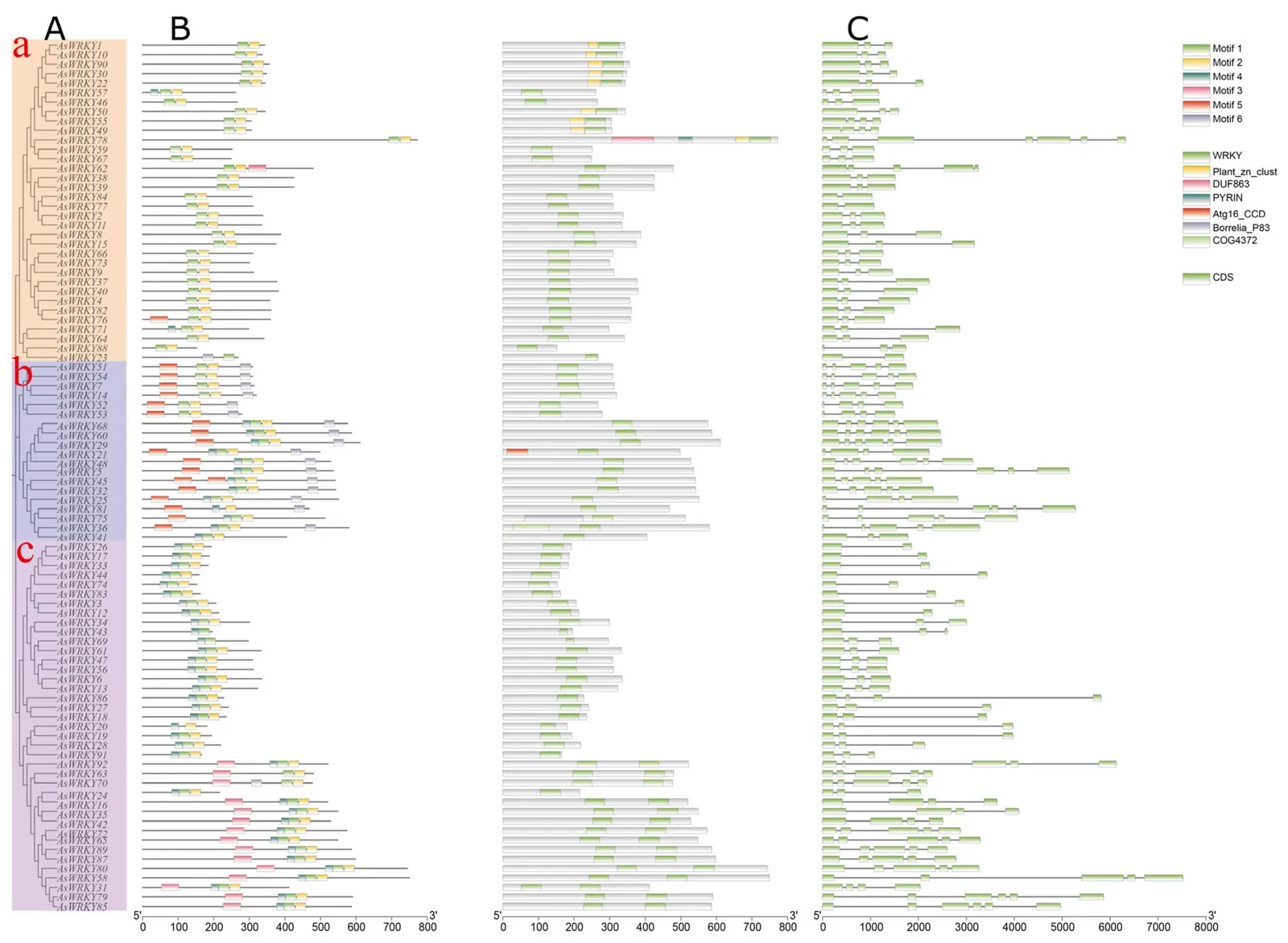
Phylogenetic relationships, distribution of cis-acting elements in promoters, and gene structure of *WRKY* gene family members in *A. sinensis.* Note: (**A**) illustrates the phylogenetic relationship of *A. sinensis*; (**a**) represents Group II, (**b**) represents Group I, and (**c**) represents Group III; (**B**) illustrates the conserved motifs; while (**C**) depicts the gene structure.

Evolutionary analysis results (Figure 4 and Figure 5A) indicated that the *WRKY* genes in *A. sinensis* are classified into three major subfamilies (Group I–Group III), each further subdivided into several smaller families. Group I (Figure 4 and Figure 5A(b)) comprised the smallest number of members (19), whereas Group III (Figure 4 and Figure 5A(c)) contained the highest number of genes, with 39 members, suggesting that *A. sinensis* may have undergone relatively significant expansion or retention within this subfamily; Group II (Figure 4 and Figure 5A(a)) follows, comprising 34 family members. The distinct branches of the three subfamilies reflected the evolutionary expansion and functional diversification of *WRKY* genes.

Six distinct conserved motifs (Motif 1–Motif 6) were identified across 92 *A. sinensis* WRKY protein sequences (Figure 5B), with variations in motif composition among different members. All WRKY proteins stably retained Motif 1, indicating that the core conserved sequence of WRKY is highly conserved across the entire gene family, consistent with the typical characteristics of the WRKY transcription factor family. Among the 92 *AsWRKY* genes, 89 genes simultaneously contained both Motif 1 and Motif 2, while *AsWRKY23*, *AsWRKY43*, and *AsWRKY69* lacked this combination, indicating that these three proteins lacked the conserved zinc finger sequence and possessed incomplete WRKY domains. It is hypothesized that sequence deletions or mutations in the zinc finger structure may lead to alterations in the protein’s spatial conformation, thereby affecting its DNA-binding capacity and transcriptional regulatory function. Furthermore, Motif 3 to Motif 6 exhibited distinct distribution patterns among family members, with varying motif combinations across different subfamilies, indicating functional divergence of the *WRKY* family during evolution. Overall, the arrangement patterns of conserved WRKY protein motifs within the same branch were highly similar, indicating a strong correlation between sequence conservation and evolutionary affinity. Figure 5C showed its genetic structure. The structural diagram revealed that genes within the same clade presented similar CDS organization. However, individual genes still varied in CDS length, indicating subtle structural variations even within the same subgroup. Such structural differences were more conspicuous among different clades.

### 3.4. Conserved Motif Analysis in A. sinensis WRKY Gene Family

**Figure 6:**
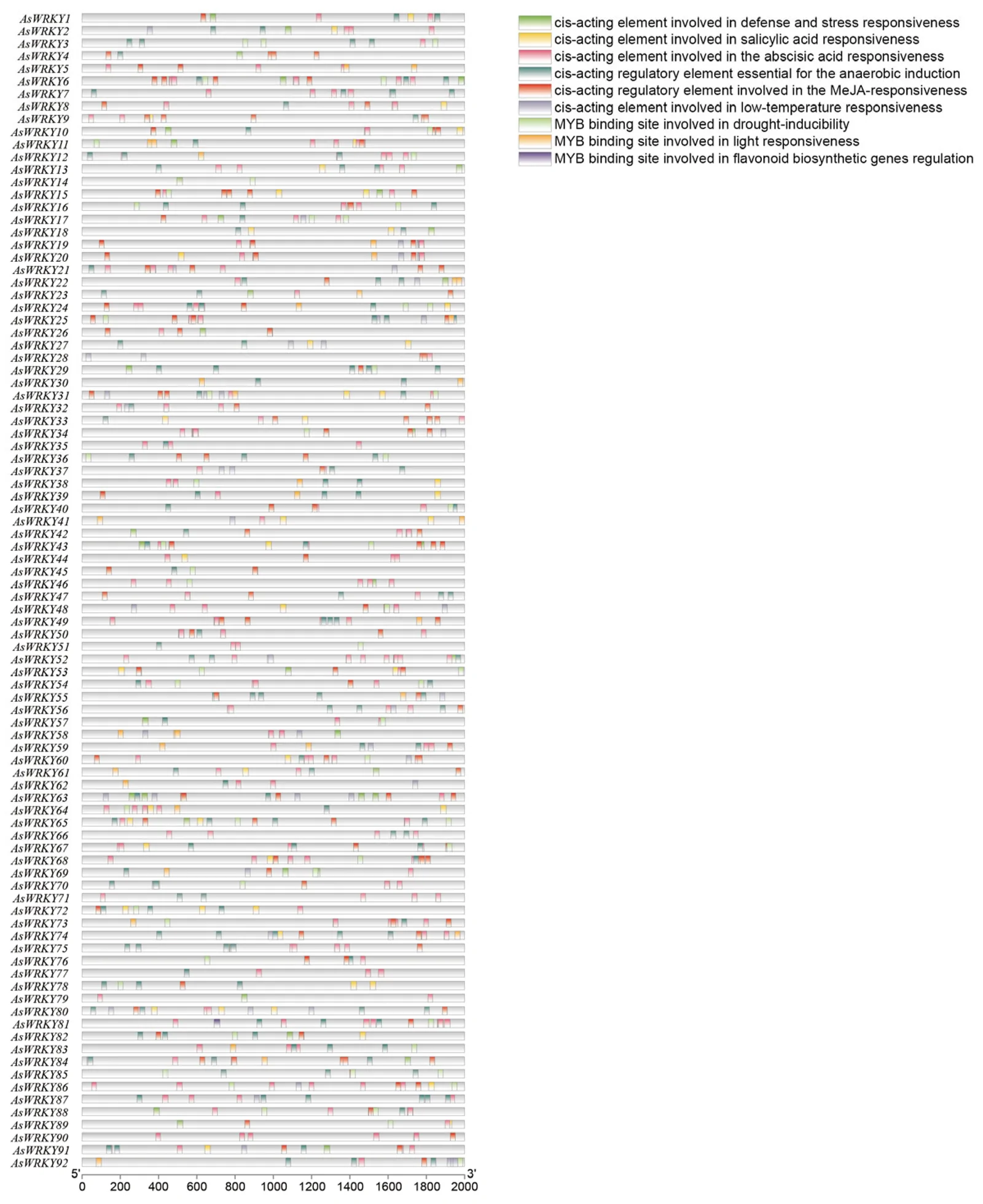
Conserved motifs of *WRKY* gene family members in *A. sinensis*.

The regulatory potential of the *AsWRKY* genes in the water stress responses of *A. sinensis* was investigated, and statistical and functional analyses were performed on the cis-regulatory elements of their promoter regions. The results demonstrated (Figure 6) that the promoter region of the *AsWRKY* gene contains multiple stress and hormone response elements, with drought-related cis-elements constituting the majority and serving as the primary functional components of this gene family under water stress conditions. These elements primarily comprised stress-defense and cold-response abiotic stress response components, as well as drought-resistant hormone-responsive elements such as salicylic acid, abscisic acid, and MeJA. They also contained numerous drought-inducible MYB binding sites, serving as the core regulatory sites mediating drought responses. In contrast, MYB binding sites associated with light response and flavonoid synthesis regulation account for a relatively small proportion, serving merely as secondary auxiliary elements. Drought-related genes such as *AsWRKY3*, *AsWRKY11*, *AsWRKY17*, *AsWRKY53*, and *AsWRKY72* exhibited differential expression under water stress conditions, suggesting they may play a pivotal role in the water stress response mechanism. In conclusion, the *AsWRKY* gene was predominantly enriched with cis-elements related to drought responses and mediated water stress regulation via multiple hormone signaling pathways and MYB-dependent mechanisms, highlighting its vital regulatory function in water metabolism in *A. sinensis*.

### 3.5. Interspecies Collinearity Analysis of WRKY Genes Between A. sinensis and Arabidopsis

**Figure 7:**
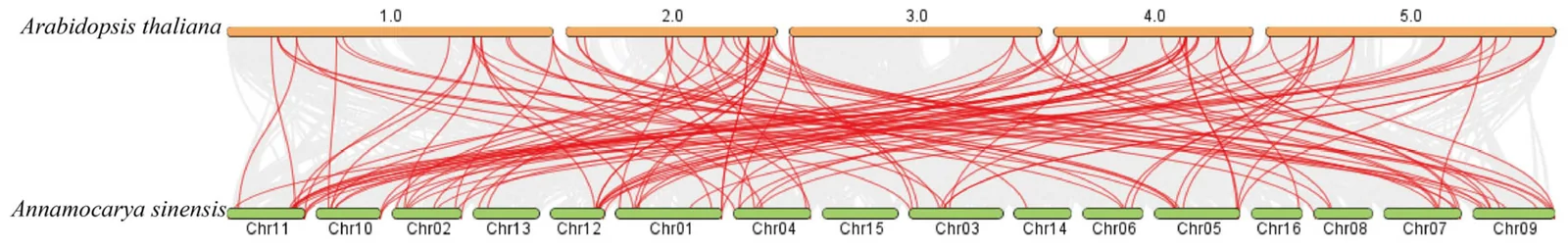
Collinearity analysis of *WRKY* genes between *A. sinensis* and *Arabidopsis thaliana*.

The inter-species collinearity analysis results (Figure 7) demonstrated a high overall genomic collinearity between *A. sinensis* and *Arabidopsis*, characterized by dense connections forming extensive continuous collinear blocks, indicating functional conservation between the two species in evolution. The regions Chr09 and Chr11 contained highly conserved collinear blocks with the strongest homologous collinearity, serving as core signaling regions. Other regions also exhibited numerous connections, and these abundant collinear blocks harbor core functional genes that are less prone to rearrangement during evolution. *AsWRKY3*, *AsWRKY11*, *AsWRKY17*, *AsWRKY53*, and *AsWRKY72* were homologous genes to *Arabidopsis thaliana*’s *AtWRKY43*, *AtWRKY22*, *AtWRKY75*, *AtWRKY40*, and *AtWRKY33*, respectively (Table 3). Among them, *AsWRKY53* was covered in Group I, *AsWRKY11* was in Group II, and *AsWRKY3*, *AsWRKY17*, and *AsWRKY72* were in Group III. Based on the known functions of homologous genes in *Arabidopsis*, their functions were inferred as positive regulation under drought stress, positive regulation under waterlogging stress, regulation under abiotic stress, positive regulation under drought stress, and positive regulation under drought stress; these five genes were subsequently selected as candidate genes for subsequent RT-qPCR experiments.

### 3.6. Expression Analysis of A. sinensis WRKY Gene Family Under Water Stress

**Figure 8:**
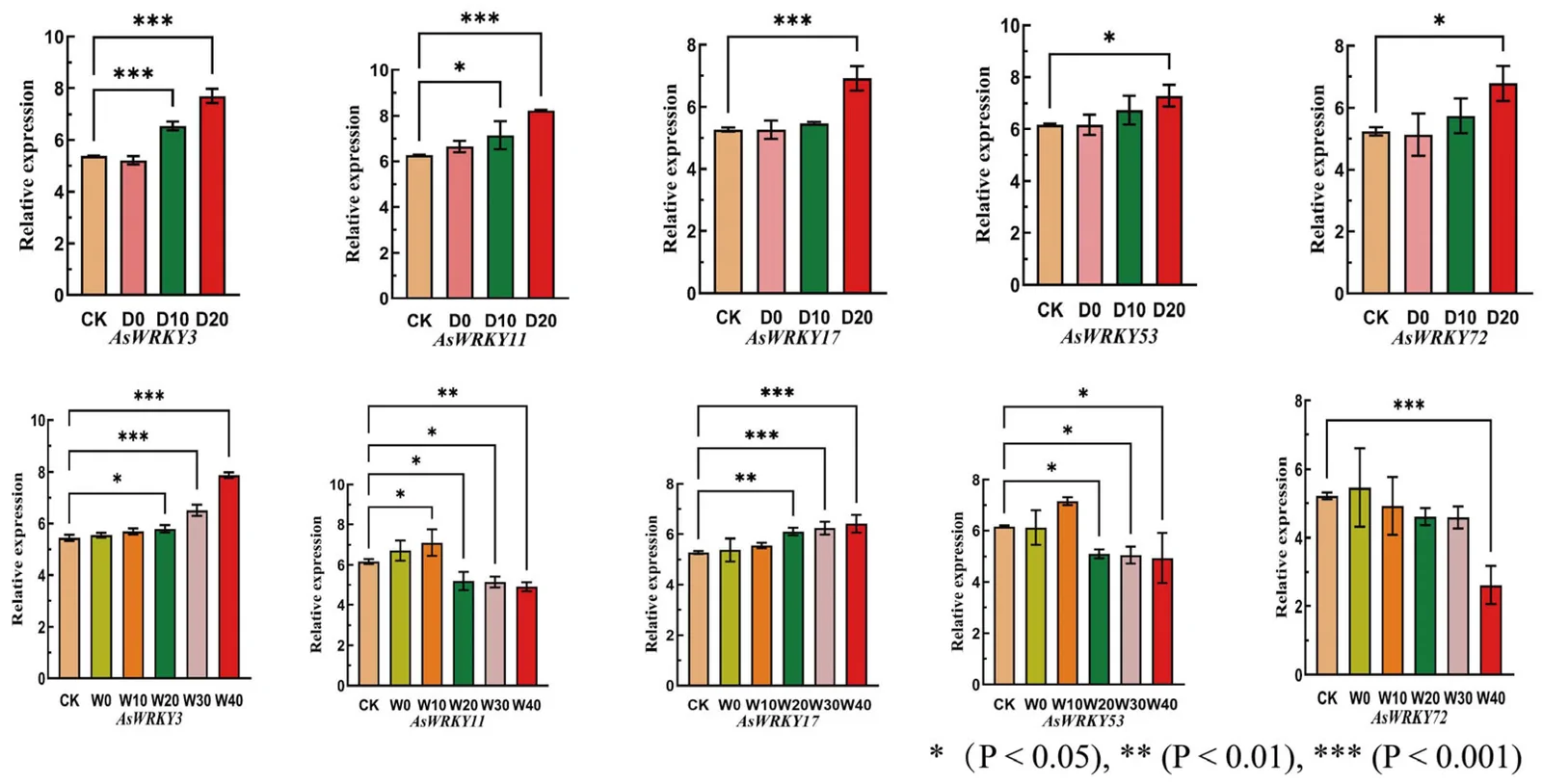
Expression levels of five genes in the *WRKY* gene family in *A.sinensis* under water stress. Note: * indicates significance (*p* < 0.05), ** indicates high significance (*p* < 0.01), *** indicates extreme significance (*p* < 0.001). Error bars represent SD, *n* = 3.

Analysis based on relative leaf water content (RWC) showed no significant difference between the control group and the drought-treated group on day 0. After 10 days of drought treatment, the leaf RWC in the drought group decreased by 32.68% compared to the control group. After 20 days of drought treatment, the RWC was reduced by 45.99% compared to the control group. To investigate the temporal variation in the expression patterns of *WRKY* genes in *A. sinensis* under water stress, five differentially expressed genes, including *AsWRKY3*, *AsWRKY11*, *AsWRKY17*, *AsWRKY53*, and *AsWRKY72*, were selected as candidates and validated via RT-qPCR. Overall, the expression patterns of the five genes varied under water stress conditions (Table 4). Under drought stress, all five genes exhibited an upregulation trend, albeit with slightly differing response magnitudes (Figure 8). *AsWRKY3*, *AsWRKY11*, and *AsWRKY17* showed highly significant differences compared to the control group at day 20, with markedly increased gene expression levels; *AsWRKY53* and *AsWRKY72* also demonstrated significant differences compared to the control group, but with less pronounced increases in expression. Under waterlogging stress, *AsWRKY3* and *AsWRKY17* exhibited strong response enhancement, *AsWRKY11* and *AsWRKY53* demonstrated early-response characteristics, and *AsWRKY72* showed a down-regulated expression trend. *AsWRKY3* and *AsWRKY17* exhibited significantly higher gene expression levels compared to the control group after a 40-day waterlogging treatment; *AsWRKY11* and *AsWRKY53* reached peak expression on day 10 of waterlogging, exhibited a strong response followed by a declining trend, indicating early-stage responses to water stress; and *AsWRKY72* showed an overall down-regulated trend, with its expression level reaching its lowest point on day 40, and exhibited the strongest response, showing a statistically significant difference from the control group. Both *AsWRKY3* and *AsWRKY17* exhibited highly significant differences compared to the control group on day 20 under drought stress and also showed highly significant differences on day 40 under waterlogging stress, demonstrating elevated relative expression levels. These two genes exhibit robust response capabilities under water stress conditions. In conclusion, *AsWRKY3*, *AsWRKY11*, *AsWRKY17*, *AsWRKY53*, and *AsWRKY72* are likely key candidate genes in the *AsWRKY* gene family of *A. sinensis* that respond to water stress. Analysis of the expression patterns of these five genes under water stress conditions demonstrated significant functional differentiation within the *WRKY* gene family during water stress response in *A. sinensis*.

## 4. Discussion

Current research on *A. sinensis* is limited, primarily focusing on breeding [4,36], seed germination characteristics and genetic diversity [37], photosynthetic properties [38], tissue culture [39], ISSR markers [38,40], whole-genome sequencing and comparative genomic analysis [41], phylogeny [42], and adaptive evolution [43]. This study characterized the *WRKY* gene family in *A. sinensis* at the genome-wide scale. A total of 92 *WRKY* genes were retrieved from the whole-genome sequence of *A. sinensis*, fewer than the 103 found in *Juglans regia* but more than the 89 observed in *Carya illinoinensis* [44,45]. This reflected differences in genomic evolution and family expansion among species. During its prolonged relict evolution, *A. sinensis* has not experienced significant family expansion compared to closely related species within the same family, likely due to its relatively conserved genome, short population history, and long-term inhabitation of stable yet fragile habitats. Transcription factor families commonly experience expansion and contraction throughout plant evolutionary processes [46]. The intergeneric discrepancy observed herein may arise from lineage-specific duplication or gene loss events occurring after the divergence of these genera. Comparative genomic investigations covering more Juglandaceae species will facilitate a better understanding of evolutionary mechanisms driving the variation in the *WRKY* gene family. These findings also underscore the species’ intrinsic genetic characteristics, which are highly consistent with its endangered status. Physicochemical property and structural analysis indicated that all 92 family members are hydrophilic proteins; this characteristic provides a reference clue for exploring their molecular behavior under water stress [47,48,49].

Analysis of cis-regulatory elements revealed that the promoter regions of *A. sinensis WRKY* genes predominantly contain drought stress-related cis-regulatory elements. All five identified candidate genes incorporate such elements, suggesting a strong association with adaptive responses to environmental stressors like water deficiency. Furthermore, numerous divergent cis-acting element types were detected, such as certain plant hormone response elements and abiotic stress response elements. This diversity of elements suggests that *AsWRKY* genes may possess varied functions.

Collinear chromosomal regions can reveal whole-genome replication events as well as relationships with chromosome replication or rearrangement [50]. Conservation analysis and evolutionary analysis revealed that the *AsWRKY* family possessed a highly conserved structural architecture, and segmental duplication was the major driving force for gene family expansion, which was consistent with the evolutionary characteristics of most plant *WRKY* families. The genomes of *A. sinensis* and *Arabidopsis* exhibited a high degree of overall genomic collinearity, characterized by dense linkage patterns that form large continuous collinear blocks. These blocks harbored core functional genes and exhibited stability against genomic rearrangement over evolutionary time. Based on the known gene functions of *Arabidopsis*, functional predictions for the five selected *A. sinensis* genes were studied and showed consistent results with those validated by RT-qPCR, further demonstrating their genetic conservation.

During the evolution process, WRKY proteins undergo multiple rounds of functional differentiation, and in response to environmental changes, most *WRKY* genes have experienced positive selection, with only a small subset subjected to negative selection [51]. Among the 89 *WRKY* genes identified in pecan, expression levels of 63 genes increased significantly, with the majority reaching peak expression levels 15 days after exposure to stress [52]. Plants deploy diverse signaling pathways to cope with abiotic stress, and stress-related genes accordingly show varied transcriptional responses [53]. Compared to deciduous trees, evergreen trees generally exhibit greater resistance under drought stress [54]. For example, in the evergreen species *Cyclobalanopsis glauca* (Fagaceae), leaf relative water content (RWC) decreases under drought stress, whereas the plants sustain physiological functions and exhibit adaptive responses [55]. *A. sinensis* is also an evergreen tree species; in the RT-qPCR verification of this experiment, *AsWRKY3*, *AsWRKY11*, *AsWRKY17*, *AsWRKY53* and *AsWRKY72* also exhibited different expression patterns during the water stress response, demonstrating significant functional differentiation and jointly participating in the water stress response process. Among them, *AsWRKY3* and *AsWRKY17* showed strong responses to both drought and waterlogging; both AsWRKY11 and *AsWRKY53* exhibited upregulation under drought stress, whereas they demonstrated early-response characteristics under waterlogging stress; specifically, *AsWRKY72* showed upregulation under drought but downregulation under waterlogging. In future molecular breeding efforts, the expression patterns of these two genes can be integrated for further research to develop superior *A. sinensis* varieties with drought and flood resistance, thereby enhancing their adaptability to environmental moisture variations. As an extremely small population, the response mechanism of *A. sinensis* to environmental stress is intrinsically linked to its survival and reproduction. The expression patterns of *WRKY* genes under drought and water stress reflect the molecular adaptation characteristics of *A. sinensis* when facing fluctuating water conditions. Analysis of such transcriptional characteristics helps us understand how this species responds to adverse hydrological environments and evaluate its adaptive potential under habitat disturbance. These findings can provide molecular references for designing targeted in situ conservation schemes, habitat restoration and artificial seedling breeding work. Furthermore, the key genes identified in this study will also provide theoretical foundations for subsequent in-depth research on gene functions and conservation efforts for endangered plant species.

Against the conservation background of *A. sinensis*, the divergent expression of *WRKY* genes under water stress provides clues for understanding how moisture conditions shape the adaptability of this endangered species [10]. Consistent up-regulation of *WRKY* genes under drought conditions and the disparate expression pattern under waterlogging conditions may imply differences in stress regulatory plasticity in response to different water conditions [10,56]. Our research findings establish a molecular association among the function of the *WRKY* gene family, water adaptability and population survival, and provide a genetic basis for promoting in situ conservation and population recovery of *A. sinensis*. Taking the candidate genes identified in this study as targets, further functional assays are expected to uncover the molecular mechanisms through which these genes participate in regulating the stress adaptation of this species.

## 5. Conclusions

This study included a whole-genome systematic analysis of the *WRKY* gene family in *A. sinensis*, revealing that the family comprises 92 members distributed unevenly across 16 chromosomes, with an overall evolutionary structure exhibiting unique species-specific characteristics. The *WRKY* domains within the family are highly conserved, with only a few members demonstrating motif deletions and structural variations, which contribute to the family’s distinct structural and functional differentiation. Evolutionary and collinearity analyses demonstrated that abundant segmental duplication sequences within the family served as the primary driver of gene family expansion and functional divergence. Moreover, the high conservation of *WRKY* homologs between *A. sinensis* and *Arabidopsis* underscored the evolutionary stability of this gene family. The prediction of promoter elements and validation of stress-induced expression results demonstrated that most *WRKY* genes are enriched with cis-element sequences responsive to drought stress and exhibited potential for the water stress response. Family members displayed distinct expression patterns under water stress conditions with significant functional differentiation, among which *AsWRKY3*, *AsWRKY11*, *AsWRKY17*, *AsWRKY53* and *AsWRKY72* represent core candidate genes involved in water stress responses. The comprehensive analysis demonstrated that the *A. sinensis WRKY* gene family exhibited both structural conservation and localized specific variations, forming a diverse water stress response mechanism through fragment replication expansion patterns, a rich array of stress response elements, and functional divergence among its members. These findings provide reliable targets for subsequent gene function validation and the elucidation of abiotic stress mechanisms, while also offering crucial genetic data support for *A. sinensis* germplasm conservation, stress-resistant gene identification, and molecular breeding efforts.

## Figures and Tables

**Table 1:** RT-qPCR primer sequences.

Gene Name	Primer Sequences (5′ → 3′)	
	Forward Primer	Reverse Primer
*AsWRKY3*	ATAACCCCACCACCTCCACT	CCAAAGGAGGAGGGTGCAAT
*AsWRKY11*	AACTCAGCCAAGCCCTCATC	TGCTGTCGATGGTTGTACCC
*AsWRKY17*	AATGGGACGACGATCTTGGG	ACCTGATCGGTTTCATCCCC
*AsWRKY53*	CGACTCTACGGACACTAGCC	GGAGCCATGGAACACCTGAA
*AsWRKY72*	TTGAACGAGCCTCCCAAGAC	AATTAGCATGGGTGCGTTGC
*Asactin*	GGTGATGCAGGGATGGTGAA	TAACGGCAAAACGACCGAGA

**Table 2:** Analysis of physicochemical properties of *WRKY* gene family members in *A. sinensis*.

Gene Number	Number of Amino Acids	Molecular Weight (kDa)	Theoretical pI	Instability Index	Aliphatic Index	Grand Average of Hydropathicity
92	151~771	17.23~86.92	5.01~9.97	26.87~67.26	42.86~72.48	−1.199~−0.445

**Table 3:** Homology alignment of candidate *AsWRKY* genes.

AsWRKY	Best Homolog (*Arabidopsis*)	Sequence Similarity	Known Function	Predicted Function
*AsWRKY3*	*AtWRKY43*	80.23%	It positively regulates gene expression, enhancing the plant’s tolerance to abiotic stresses such as drought [27,28].	Positive regulation under drought stress.
*AsWRKY11*	*AtWRKY22*	75.61%	Enhances flood resistance and activates transcription factors [29,30].	Waterlogging stress is regulated positively.
*AsWRKY17*	*AtWRKY75*	74.31%	Saline stress promotes gene expression and influences leaf senescence [31,32].	Regulation of abiotic stress.
*AsWRKY53*	*AtWRKY40*	75.36%	Regulation of drought stress response and defense mechanisms [33,34].	Positive regulation under drought stress.
*AsWRKY72*	*AtWRKY33*	70.82%	Resists coercion, such as drought [33,35].	Positive regulation under drought stress.

**Table 4:** Expression patterns of *WRKY* family genes of *A. sinensis* under different water stresses.

Drought Stress	Expression Mode	Waterlogging Stress	Expression Mode
*AsWRKY3*	Positive Regulation (***)	*AsWRKY3*	Positive Regulation (***)
*AsWRKY11*	Positive Regulation (***)	*AsWRKY11*	Early Response (**)
*AsWRKY17*	Positive Regulation (***)	*AsWRKY17*	Positive Regulation (***)
*AsWRKY53*	Weak Response (***)	*AsWRKY53*	Early Response (*)
*AsWRKY72*	Weak Response (*)	*AsWRKY72*	Negative Regulation (***)

Note: * indicates significance (*p* < 0.05), ** indicates high significance (*p* < 0.01), *** indicates extreme significance (*p* < 0.001).

## Data Availability

The data are contained within this manuscript and the Supplementary Materials.

## Supplementary Materials

The following supporting information can be downloaded at https://www.mdpi.com/article/10.3390/biology15161429/s1: Table S1: Analysis of physicochemical properties of *WRKY* genes in *A. sinensis*; Table S2: Gene IDs of *WRKY* genes from *A. sinensis* and *Arabidopsis thaliana*.

## Abbreviations

The following abbreviations are used in this manuscript:

RT-qPCR	Reverse Transcription Quantitative PCR
*As*	*Annamocarya sinensis*
TAIR	The Arabidopsis Information Resource
NCBI	National Center for Biotechnology Information
BLAST	Basic Local Alignment Search Tool
HMM	Hidden Markov Model
Pfam	Protein Families Database
MEGA	Molecular Evolutionary Genetics Analysis
CD-search	Conserved Domain Search
*Arabidopsis*	*Arabidopsis thaliana*
MEME	Motif-Based Sequence Analysis Tools
bp	Base Pair
Chr	Chromosome
CDS	Coding Sequence
MeJA	Methyl Jasmonate
SD	Standard Deviation
