# Supplementary material for: Genome-Wide Identification and Water Stress Response of the WRKY Gene Family in Annamocarya sinensis: An Endangered Plant with an Extremely Small Population
Source: Biology (Basel). 2026 Aug 19;15(16):1429. doi: 10.3390/biology15161429 (PMC13509280; doi:10.3390/biology15161429)
Supplement: Supplementary file 1 [file biology-15-01429-s001.zip › Table S1. Analysis of physicochemical properties of WRKY genes in A. sinensisdocx.pdf]

Table S1. Analysis of physicochemical properties of *WRKY* genes in *A. sinensis*

Sequence ID	Number of Amino Acid	Molecular Weight(KDa)	Theoretical pI	Instability Index	Aliphatic Index	Grand Average of Hydropathicity
AsWRKY1	342	37.48	9.58	55.17	65.03	-0.651
AsWRKY2	337	36.97	5.57	57.08	50.15	-0.812
AsWRKY3	205	23.46	9.54	49.00	65.61	-0.689
AsWRKY4	356	39.90	5.28	52.61	58.06	-0.745
AsWRKY5	535	58.94	6.70	52.89	68.28	-0.591
AsWRKY6	334	37.63	5.95	67.06	47.31	-0.987
AsWRKY7	312	34.52	8.39	46.95	63.14	-0.779
AsWRKY8	387	42.48	5.96	62.95	64.50	-0.674
AsWRKY9	311	35.10	5.35	57.87	63.05	-0.722
AsWRKY10	335	37.01	9.51	41.60	66.66	-0.621
AsWRKY11	334	36.50	5.20	59.49	53.47	-0.765
AsWRKY12	213	24.38	8.97	48.89	66.76	-0.708
AsWRKY13	322	36.09	7.64	64.36	51.46	-0.877
AsWRKY14	318	35.30	9.00	41.96	59.21	-0.873
AsWRKY15	374	41.50	6.77	57.86	55.86	-0.755
AsWRKY16	519	56.62	7.26	60.04	58.50	-0.765
AsWRKY17	186	21.59	9.45	38.00	53.39	-0.928
AsWRKY18	234	26.18	8.78	47.37	67.95	-0.585
AsWRKY19	193	21.63	5.81	47.86	57.05	-0.802
AsWRKY20	180	20.17	5.03	49.15	59.56	-0.718
AsWRKY21	497	53.61	8.14	41.48	61.33	-0.669
AsWRKY22	343	38.04	9.60	58.62	70.20	-0.667
AsWRKY23	267	28.58	6.42	65.88	48.35	-0.570
AsWRKY24	215	23.68	7.76	47.49	63.49	-1.010
AsWRKY25	550	60.38	8.44	50.81	61.05	-0.692
AsWRKY26	192	21.87	9.42	44.96	57.29	-0.770
AsWRKY27	240	27.17	9.22	53.50	57.25	-0.810
AsWRKY28	218	24.82	6.63	51.00	52.29	-1.061
AsWRKY29	610	66.10	6.51	46.10	63.59	-0.603
AsWRKY30	347	39.10	9.52	53.36	63.49	-0.815
AsWRKY31	410	45.66	6.06	44.57	60.24	-0.853
AsWRKY32	541	59.59	6.16	43.54	68.19	-0.737
AsWRKY33	183	20.80	9.47	43.07	54.32	-0.859
AsWRKY34	299	33.06	5.79	64.13	44.65	-0.966
AsWRKY35	548	59.21	8.09	54.38	54.85	-0.840
AsWRKY36	579	62.50	6.48	47.15	64.44	-0.609
AsWRKY37	376	41.97	6.18	63.46	58.91	-0.717
AsWRKY38	424	46.56	6.47	67.26	51.84	-0.925
AsWRKY39	424	46.55	6.33	66.30	51.84	-0.924
AsWRKY40	380	42.45	5.70	57.43	56.97	-0.728

Sequence ID	Number of Amino Acid	Molecular Weight(KDa)	Theoretical pI	Instability Index	Aliphatic Index	Grand Average of Hydropathicity
AsWRKY41	404	44.89	8.98	51.91	59.88	-0.784
AsWRKY42	527	57.03	7.70	53.71	59.24	-0.733
AsWRKY43	195	21.50	5.45	57.07	57.49	-0.793
AsWRKY44	158	18.17	9.64	43.93	49.94	-1.085
AsWRKY45	540	60.22	5.84	45.30	68.30	-0.777
AsWRKY46	265	30.13	5.51	48.99	61.43	-0.884
AsWRKY47	308	34.27	8.19	61.81	60.52	-0.677
AsWRKY48	527	57.90	8.15	52.35	58.75	-0.743
AsWRKY49	305	33.42	9.69	54.05	62.36	-0.644
AsWRKY50	343	36.83	9.67	42.95	65.95	-0.445
AsWRKY51	308	34.61	8.37	53.44	65.88	-0.717
AsWRKY52	266	29.95	7.12	56.56	67.14	-0.781
AsWRKY53	278	31.07	8.54	57.25	72.27	-0.671
AsWRKY54	308	34.58	7.62	48.05	65.29	-0.671
AsWRKY55	304	33.55	9.74	46.03	68.03	-0.570
AsWRKY56	310	34.50	8.92	62.50	60.39	-0.685
AsWRKY57	260	29.77	6.10	53.97	67.08	-0.963
AsWRKY58	749	81.83	5.52	45.76	68.62	-0.555
AsWRKY59	250	27.78	5.30	58.90	53.40	-0.739
AsWRKY60	586	63.66	6.22	48.40	65.00	-0.605
AsWRKY61	332	36.27	6.66	63.96	45.57	-0.778
AsWRKY62	478	51.54	5.28	48.19	54.92	-0.549
AsWRKY63	479	52.07	8.90	49.86	58.37	-0.824
AsWRKY64	340	38.47	5.57	56.09	59.44	-0.902
AsWRKY65	547	60.98	6.82	64.12	43.49	-0.959
AsWRKY66	309	34.90	6.00	62.94	70.39	-0.692
AsWRKY67	248	27.78	5.05	54.45	53.47	-0.804
AsWRKY68	575	61.83	6.26	45.52	63.37	-0.613
AsWRKY69	296	33.36	6.46	57.68	49.16	-0.876
AsWRKY70	476	51.76	9.11	45.01	54.26	-0.904
AsWRKY71	297	33.50	5.09	54.17	70.98	-0.635
AsWRKY72	573	63.86	6.07	53.32	48.17	-0.916
AsWRKY73	299	33.88	5.30	59.67	72.44	-0.583
AsWRKY74	152	17.55	9.56	48.59	50.66	-1.114
AsWRKY75	512	56.81	6.45	55.70	49.41	-0.931
AsWRKY76	358	40.27	5.41	60.83	66.45	-0.640
AsWRKY77	309	34.73	5.85	60.71	64.69	-0.596
AsWRKY78	771	86.92	8.40	59.93	72.48	-0.688
AsWRKY79	589	64.24	7.07	47.19	64.02	-0.677
AsWRKY80	743	79.91	5.74	59.60	58.94	-0.697
AsWRKY81	467	52.15	8.73	48.09	66.60	-0.719
AsWRKY82	360	40.27	5.76	52.25	60.67	-0.647

Sequence ID	Number of Amino Acid	Molecular Weight(KDa)	Theoretical pI	Instability Index	Aliphatic Index	Grand Average of Hydropathicity
AsWRKY83	161	18.32	9.97	26.87	64.78	-0.804
AsWRKY84	307	34.33	7.01	63.73	63.49	-0.668
AsWRKY85	586	63.40	6.93	45.23	60.22	-0.714
AsWRKY86	227	25.63	6.99	44.31	42.86	-1.004
AsWRKY87	597	65.68	7.34	60.82	47.40	-0.935
AsWRKY88	151	17.23	6.48	42.57	48.41	-1.199
AsWRKY89	586	64.35	6.41	57.58	45.00	-1.010
AsWRKY90	355	38.22	9.26	56.88	71.46	-0.449
AsWRKY91	165	19.01	5.01	56.63	47.76	-1.170
AsWRKY92	520	57.17	5.52	66.49	59.29	-0.973
