# Supplementary material for: Genome-Wide Identification and Water Stress Response of the WRKY Gene Family in Annamocarya sinensis: An Endangered Plant with an Extremely Small Population
Source: Biology (Basel). 2026 Aug 19;15(16):1429. doi: 10.3390/biology15161429 (PMC13509280; doi:10.3390/biology15161429)
Supplement: Supplementary file 1 [file biology-15-01429-s001.zip › Table S2. Gene IDs of WRKY genes from A. sinensis and Arabidopsis thaliana.pdf]

Table S2. Gene IDs of *WRKY* genes from *A. sinensis* and *Arabidopsis thaliana.*

Species	Gene name	Gene ID
Annamocarya sinensis	*AsWRKY1*	*ASI030166.1*
Annamocarya sinensis	*AsWRKY2*	*ASI031669.1*
Annamocarya sinensis	*AsWRKY3*	*ASI031651.1*
Annamocarya sinensis	*AsWRKY4*	*ASI017998.1*
Annamocarya sinensis	*AsWRKY5*	*ASI020309.1*
Annamocarya sinensis	*AsWRKY6*	*ASI020348.1*
Annamocarya sinensis	*AsWRKY7*	*ASI011256.1*
Annamocarya sinensis	*AsWRKY8*	*ASI011549.1*
Annamocarya sinensis	*AsWRKY9*	*ASI011621.1*
Annamocarya sinensis	*AsWRKY10*	*ASI031140.1*
Annamocarya sinensis	*AsWRKY11*	*ASI023786.1*
Annamocarya sinensis	*AsWRKY12*	*ASI023695.1*
Annamocarya sinensis	*AsWRKY13*	*ASI009499.1*
Annamocarya sinensis	*AsWRKY14*	*ASI006557.1*
Annamocarya sinensis	*AsWRKY15*	*ASI033321.1*
Annamocarya sinensis	*AsWRKY16*	*ASI002795.1*
Annamocarya sinensis	*AsWRKY17*	*ASI003218.1*
Annamocarya sinensis	*AsWRKY18*	*ASI026982.1*
Annamocarya sinensis	*AsWRKY19*	*ASI027032.1*
Annamocarya sinensis	*AsWRKY20*	*ASI015511.1*
Annamocarya sinensis	*AsWRKY21*	*ASI015504.1*
Annamocarya sinensis	*AsWRKY22*	*ASI001978.1*
Annamocarya sinensis	*AsWRKY23*	*ASI025316.1*
Annamocarya sinensis	*AsWRKY24*	*ASI025352.1*
Annamocarya sinensis	*AsWRKY25*	*ASI025176.1*
Annamocarya sinensis	*AsWRKY26*	*ASI029854.1*
Annamocarya sinensis	*AsWRKY27*	*ASI012605.1*
Annamocarya sinensis	*AsWRKY28*	*ASI012724.1*
Annamocarya sinensis	*AsWRKY29*	*ASI021237.1*
Annamocarya sinensis	*AsWRKY30*	*ASI028697.1*
Annamocarya sinensis	*AsWRKY31*	*ASI032383.1*
Annamocarya sinensis	*AsWRKY32*	*ASI020875.1*
Annamocarya sinensis	*AsWRKY33*	*ASI001235.1*
Annamocarya sinensis	*AsWRKY34*	*ASI001884.1*
Annamocarya sinensis	*AsWRKY35*	*ASI001492.1*
Annamocarya sinensis	*AsWRKY36*	*ASI001397.1*
Annamocarya sinensis	*AsWRKY37*	*ASI009577.1*
Annamocarya sinensis	*AsWRKY38*	*ASI009846.1*
Annamocarya sinensis	*AsWRKY39*	*ASI033242.1*
Annamocarya sinensis	*AsWRKY40*	*ASI012800.1*
Annamocarya sinensis	*AsWRKY41*	*ASI016789.1*

Annamocarya sinensis	*AsWRKY42*	*ASI016866.1*

Species	Gene name	Gene ID
Annamocarya sinensis	*AsWRKY43*	*ASI016751.1*
Annamocarya sinensis	*AsWRKY44*	*ASI026697.1*
Annamocarya sinensis	*AsWRKY45*	*ASI022764.1*
Annamocarya sinensis	*AsWRKY46*	*ASI007719.1*
Annamocarya sinensis	*AsWRKY47*	*ASI008003.1*
Annamocarya sinensis	*AsWRKY48*	*ASI007862.1*
Annamocarya sinensis	*AsWRKY49*	*ASI010150.1*
Annamocarya sinensis	*AsWRKY50*	*ASI010386.1*
Annamocarya sinensis	*AsWRKY51*	*ASI010379.1*
Annamocarya sinensis	*AsWRKY52*	*ASI010154.1*
Annamocarya sinensis	*AsWRKY53*	*ASI032567.1*
Annamocarya sinensis	*AsWRKY54*	*ASI032500.1*
Annamocarya sinensis	*AsWRKY55*	*ASI016578.1*
Annamocarya sinensis	*AsWRKY56*	*ASI012216.1*
Annamocarya sinensis	*AsWRKY57*	*ASI012097.1*
Annamocarya sinensis	*AsWRKY58*	*ASI029783.1*
Annamocarya sinensis	*AsWRKY59*	*ASI026233.1*
Annamocarya sinensis	*AsWRKY60*	*ASI020019.1*
Annamocarya sinensis	*AsWRKY61*	*ASI007311.1*
Annamocarya sinensis	*AsWRKY62*	*ASI025826.1*
Annamocarya sinensis	*AsWRKY63*	*ASI004044.1*
Annamocarya sinensis	*AsWRKY64*	*ASI004060.1*
Annamocarya sinensis	*AsWRKY65*	*ASI027770.1*
Annamocarya sinensis	*AsWRKY66*	*ASI027708.1*
Annamocarya sinensis	*AsWRKY67*	*ASI005665.1*
Annamocarya sinensis	*AsWRKY68*	*ASI005903.1*
Annamocarya sinensis	*AsWRKY69*	*ASI005701.1*
Annamocarya sinensis	*AsWRKY70*	*ASI006980.1*
Annamocarya sinensis	*AsWRKY71*	*ASI007088.1*
Annamocarya sinensis	*AsWRKY72*	*ASI029372.1*
Annamocarya sinensis	*AsWRKY73*	*ASI034033.1*
Annamocarya sinensis	*AsWRKY74*	*ASI013639.1*
Annamocarya sinensis	*AsWRKY75*	*ASI015228.1*
Annamocarya sinensis	*AsWRKY76*	*ASI009126.1*
Annamocarya sinensis	*AsWRKY77*	*ASI005000.1*
Annamocarya sinensis	*AsWRKY78*	*ASI004653.1*
Annamocarya sinensis	*AsWRKY79*	*ASI004385.1*
Annamocarya sinensis	*AsWRKY80*	*ASI004982.1*
Annamocarya sinensis	*AsWRKY81*	*ASI013934.1*
Annamocarya sinensis	*AsWRKY82*	*ASI000038.1*
Annamocarya sinensis	*AsWRKY83*	*ASI001010.1*
Annamocarya sinensis	*AsWRKY84*	*ASI000692.1*

Annamocarya sinensis	*AsWRKY85*	*ASI029635.1*

Species	Gene name	Gene ID
Annamocarya sinensis	*AsWRKY86*	*ASI017321.1*
Annamocarya sinensis	*AsWRKY87*	*ASI018283.1*
Annamocarya sinensis	*AsWRKY88*	*ASI023622.1*
Annamocarya sinensis	*AsWRKY89*	*ASI011815.1*
Annamocarya sinensis	*AsWRKY90*	*ASI014176.1*
Annamocarya sinensis	*AsWRKY91*	*ASI032054.1*
Annamocarya sinensis	*AsWRKY92*	*ASI033482.1*
Arabidopsis thaliana	*AtWRKY1*	*AT2G04880*
Arabidopsis thaliana	*AtWRKY2*	*AT5G56270*
Arabidopsis thaliana	*AtWRKY3*	*AT2G03340*
Arabidopsis thaliana	*AtWRKY4*	*AT1G13960*
Arabidopsis thaliana	*AtWRKY6*	*AT1G62300*
Arabidopsis thaliana	*AtWRKY7*	*AT4G24240*
Arabidopsis thaliana	*AtWRKY8*	*AT5G46350*
Arabidopsis thaliana	*AtWRKY9*	*AT1G68150*
Arabidopsis thaliana	*AtWRKY10*	*AT1G55600*
Arabidopsis thaliana	*AtWRKY11*	*AT4G31550*
Arabidopsis thaliana	*AtWRKY12*	*AT2G44745*
Arabidopsis thaliana	*AtWRKY13*	*AT4G39410*
Arabidopsis thaliana	*AtWRKY14*	*AT1G30650*
Arabidopsis thaliana	*AtWRKY15*	*AT2G23320*
Arabidopsis thaliana	*AtWRKY16*	*AT5G45050*
Arabidopsis thaliana	*AtWRKY17*	*AT2G24570*
Arabidopsis thaliana	*AtWRKY18*	*AT4G31800*
Arabidopsis thaliana	*AtWRKY19*	*AT4G12020*
Arabidopsis thaliana	*AtWRKY20*	*AT4G26640*
Arabidopsis thaliana	*AtWRKY21*	*AT2G30590*
Arabidopsis thaliana	*AtWRKY22*	*AT4G01250*
Arabidopsis thaliana	*AtWRKY23*	*AT2G47260*
Arabidopsis thaliana	*AtWRKY24*	*AT5G41570*
Arabidopsis thaliana	*AtWRKY25*	*AT2G30250*
Arabidopsis thaliana	*AtWRKY26*	*AT5G07100*
Arabidopsis thaliana	*AtWRKY27*	*AT5G52830*
Arabidopsis thaliana	*AtWRKY28*	*AT4G18170*
Arabidopsis thaliana	*AtWRKY29*	*AT4G23550*
Arabidopsis thaliana	*AtWRKY30*	*AT5G24110*
Arabidopsis thaliana	*AtWRKY31*	*AT4G22070*
Arabidopsis thaliana	*AtWRKY32*	*AT4G30935*
Arabidopsis thaliana	*AtWRKY33*	*AT2G38470*
Arabidopsis thaliana	*AtWRKY34*	*AT4G26440*
Arabidopsis thaliana	*AtWRKY35*	*AT2G34830*
Arabidopsis thaliana	*AtWRKY36*	*AT1G69810*

Species	Gene name	Gene ID
Arabidopsis thaliana	*AtWRKY38*	*AT5G22570*
Arabidopsis thaliana	*AtWRKY39*	*AT3G04670*
Arabidopsis thaliana	*AtWRKY40*	*AT1G80840*
Arabidopsis thaliana	*AtWRKY41*	*AT4G11070*
Arabidopsis thaliana	*AtWRKY42*	*AT4G04450*
Arabidopsis thaliana	*AtWRKY43*	*AT2G46130*
Arabidopsis thaliana	*AtWRKY44*	*AT2G37260*
Arabidopsis thaliana	*AtWRKY45*	*AT3G01970*
Arabidopsis thaliana	*AtWRKY46*	*AT2G46400*
Arabidopsis thaliana	*AtWRKY47*	*AT4G01720*
Arabidopsis thaliana	*AtWRKY48*	*AT5G49520*
Arabidopsis thaliana	*AtWRKY49*	*AT5G43290*
Arabidopsis thaliana	*AtWRKY50*	*AT5G26170*
Arabidopsis thaliana	*AtWRKY51*	*AT5G64810*
Arabidopsis thaliana	*AtWRKY52*	*AT5G45260*
Arabidopsis thaliana	*AtWRKY53*	*AT4G23810*
Arabidopsis thaliana	*AtWRKY54*	*AT2G40750*
Arabidopsis thaliana	*AtWRKY55*	*AT2G40740*
Arabidopsis thaliana	*AtWRKY56*	*AT1G64000*
Arabidopsis thaliana	*AtWRKY57*	*AT1G69310*
Arabidopsis thaliana	*AtWRKY58*	*AT3G01080*
Arabidopsis thaliana	*AtWRKY59*	*AT2G21900*
Arabidopsis thaliana	*AtWRKY60*	*AT2G25000*
Arabidopsis thaliana	*AtWRKY61*	*AT1G18860*
Arabidopsis thaliana	*AtWRKY62*	*AT5G01900*
Arabidopsis thaliana	*AtWRKY63*	*AT1G66600*
Arabidopsis thaliana	*AtWRKY64*	*AT1G66560*
Arabidopsis thaliana	*AtWRKY65*	*AT1G29280*
Arabidopsis thaliana	*AtWRKY66*	*AT1G80590*
Arabidopsis thaliana	*AtWRKY67*	*AT1G66550*
Arabidopsis thaliana	*AtWRKY68*	*AT3G62340*
Arabidopsis thaliana	*AtWRKY69*	*AT3G58710*
Arabidopsis thaliana	*AtWRKY70*	*AT3G56400*
Arabidopsis thaliana	*AtWRKY71*	*AT1G29860*
Arabidopsis thaliana	*AtWRKY72*	*AT5G15130*
Arabidopsis thaliana	*AtWRKY74*	*AT5G28650*
Arabidopsis thaliana	*AtWRKY75*	*AT5G13080*
